# Adaptation and Validation of the Chinese Version of the Nutrition Environment Measurement Tool for Stores

**DOI:** 10.3390/ijerph16050782

**Published:** 2019-03-04

**Authors:** Yang Liu, Shenzhi Song, Joel Gittelsohn, Nan Jiang, Jiajin Hu, Yanan Ma, Deliang Wen

**Affiliations:** 1 Institute of Health Sciences, China Medical University, Shenyang 110122, China; yliu0568@cmu.edu.cn (Y.L.); szsong@cmu.edu.cn (S.S.); ndjiang@live.ca (N.J.); jjhu@cmu.edu.cn (J.H.); ynma@cmu.edu.cn (Y.M.); 2 Department of International Health, Center for Human Nutrition, Johns Hopkins Bloomberg School of Public Health, Baltimore, MD 21205-2179, USA; jgittel1@jhu.edu

**Keywords:** food environment, China, Nutritional Environment Measurement Survey-Stores (NEMS-S)

## Abstract

Changes in lifestyle and food environment have created a heavy burden of obesity and chronic disease in China. However, measurements of the food environment have been rarely reported in China or other countries with similar food cultures; this measurement shortage is partially due to the lack of valid and reliable measurement tools. The aim of the present study was to adapt and validate a Chinese version of the Nutritional Environment Measurement Survey for Stores (C-NEMS-S). Categories and items of the NEMS-S were culturally adapted to fit the Chinese population and included grains, dry beans, starchy tubers, vegetables, fruits, seafood, meat and poultry, dietary oils, milk, bread, instant noodles, and beverages. A scoring sheet for each food category was created to measure availability, quality, and pricing. Then, the C-NEMS-S was validated in 10 large-sized supermarkets and 10 convenience stores in Shenyang, China. Two trained raters performed their evaluations separately at the same store. The intra-class correlation coefficient (ICC) of the availability composite score was 0.98. All food measures had a moderate or good ICC (0.41 to 1.00). The kappa for each food measure ranged from 0.52 to 1.00. C-NEMS-S was able to show the difference in healthy food availability between large-sized supermarkets and convenience stores, as well as the price differences between healthier options and regular options. Large-sized supermarkets had a significantly higher total score (*p* < 0.001) and healthier option availability for all food measures (all items were statistically significant (*p* < 0.05), except sugar-free beverages). Healthier options cost more than regular options for grains, milk, bread, and instant noodles (from 4% to 153%). The adapted C-NEMS-S can be used to measure the consumer food environment in stores in China.

## 1. Introduction

The food environment, which could also be called a nutrition environment, refers to the physical presence of food that affects a person’s diet, a person’s proximity to food store locations, the distribution of food stores, food service, any physical entity by which food may be obtained, or a connected system that allows access to food [1]. The food environment plays an important role in food choice, eating patterns, and energy intake [2]. It is widely reported that the food environment is associated with the increasing epidemic of childhood and adult obesity [3,4,5,6]. Recent efforts on obesity prevention have partly focused on studying the role of environmental factors, and national level policies on the food environment have been implemented in many countries [7,8,9].

It was reported that the food environment within stores, as it relates to healthy food availability, quality, and price, may contribute to the dietary intake disparities in many countries [10,11,12]. Specifically, higher quality food stores, such as specialized fruit and vegetable (F&V) markets, were associated with greater F&V intake after controlling for individual-level characteristics, according to a study conducted in a Brazilian city [10]. The proportion of in-store shelf space for skim, 1% fat, and 2% fat milk was reported by a study conducted in the United States to be associated with low-fat milk consumption [11]. Besides price and convenience, purity, freshness, association with specific places, and ‘Pakistani-ness’ were reported as the bases for making decisions about ‘good food’ in Pakistan [12].

Over the past several decades, China has been undergoing economic development and urbanization at an accelerated rate [13]. In 1950, 13% of people in China lived in cities. By 2010, the urban share of the population had grown to 45%; this is projected to reach 60% by 2030 [14]. Meanwhile, the total energy intake of Chinese people increased by 80%, from 1635 kcal/capita/d in 1961 to 2943 kcal/capita/d in 2003. By 2006, less than 1 percent of people were getting 10 percent of their energy from fat, 44 percent were getting 30 percent of their energy from fat, and nearly two-thirds were getting more than 10 percent of their energy from animal products [15]. The prevalence of overweight and obese children increased from less than 2% in 1985 to 15% in 2010, and the overall rate of overweight and obese people in major cities is over 20% [16,17,18]. However, the food environment after such huge societal change in China, and its contribution to the obesity prevalence in China, has yet to be described. Answers these questions would help provide evidence for food environment improvement and potentially limit the obesity epidemic in China.

Lack of valid, reliable measures of food environments hinders food environment studies in China and other Asian countries. Many food environment measurement tools exist for other countries [19,20]. Among these, the Nutritional Environment Measures Survey in Stores (NEMS-S) is an observational measure to assess the food environment within retail food stores [21,22]. The NEMS-S measures the availability of healthier options, price, and quality for 10 categories of food products. The NEMS-S is characterized by its relative ease of use and ability to adapt to different settings and populations [23,24,25,26,27]. The 10 categories of food products included in the NEMS-S were based on the types of food products that contributed the most fat and calories, which are different from those in China, to the American diet. So, before using the NEMS-S in China, food products were culturally adapted in this study to fit the Chinese food culture. This study aimed to adapt and validate a Chinese version of the Nutritional Environment Measurement Survey for Stores (C-NEMS-S) in order to provide a tool for measuring the food environment in China for future studies.

## 2. Methods

### 2.1. Adaptation of Food Measures and Healthier Options

The basic principle of the NEMS-S is to gather information on comparable categories and species of food across stores, which includes the availability, price, and quality of healthy options for a particular food. Three steps were conducted to adapt the NEMS-S to the C-NEMS-S: selecting food measures, defining healthy food or healthier options, and adapting scores for availability, price, and quality.

#### 2.1.1. Selection of Food Measures

Food measures were selected based on the foods that contribute the most energy to the Chinese diet, as listed in the China Food Composition handbook developed by The Institute of Nutrition and Food Safety at the National Centers for Disease Control and Prevention [28] and in the Chinese Dietary Guidelines developed by the Chinese Nutrition Society, Ministry of Health, Ministry of Agriculture, General Administration of Sport, and National Centers for Disease Control and Prevention [29].

Chinese people divided formal meals into two general parts: “fan” and “cai” [2]. “Fan” is the staple food for every Chinese meal, accounts for a main part of Chinese dietary energy intake, and comprises grains (such as rice, wheat, maize, millet, or sorghum), dry beans, starchy tubers, or root vegetables (such as potatoes, cassava, yams, or taro) [3,29]. It was reported that, by 2015, annual consumption of grains in China had risen to 534 kg per person [30]. Hence, grains, dry beans, and starchy tubers were listed into the C-NEMS-S as the first three food categories. “Cai” could be made of various kinds of vegetables, meat and poultry, and dietary oil. Eight subcategories of vegetables (root vegetables; fresh beans; solanaceous fruit vegetables; bulb vegetables; tender stems, leaves and cauliflower vegetables; aquatic vegetables; wild vegetables; bacteria and algae vegetables) were included into the C-NEMS-S, according to the China Food Composition handbook, which covers all kinds of vegetables eaten by the Chinese people [28]. Meat and poultry that could be cooked as dishes for meals in China included poultry and livestock (pork, beef, mutton, chicken, duck, etc.) and seafood (fish, shrimp, crab, shellfish: freshwater or saltwater). Another important ingredient in Chinese meals was dietary oil, of which Chinese residents consume an average of 42.1 kg per year per person; dietary oil was reported to be related to nutrition-related non-communicable diseases (NR-NCDs) among the poor [31].

Breads and instant noodles are the foods most often consumed as instant meals, according to the China Food Composition handbook and were included in the C-NEMS-S.

Milk is being consumed more frequently in China, with the rise of household income [32]. Milk was recommended by the Chinese Food Guide as a healthy food item. Milk’s measurements and scoring in the C-NEMS-S were kept the same as in the original NEMS-S.

Six subcategories of fruits (pip fruits, stone fruits, soft fruits, citrus fruits, subtropical and tropical fruits, and melons) were included into the C-NEMS-S and cover all kinds of fruits consumed in China [28].

Six subcategories of beverages (lactic acid drinks, milk beverages, juices, teas, plant protein beverages, apple vinegar) were included into the C-NEMS-S and cover all kinds of beverages consumed in China [28].

#### 2.1.2. Definition of Healthy Foods or Healthier Options

An important principle of the NEMS-S was to measure availability, price, and quality of “healthier” options (such as low-fat milk for the milk measure and diet coke for the beverage measure) or healthy foods (such as fruits or vegetables). Healthier options and healthy foods are defined and presented in Table 1.

#### 2.1.3. Adaptation of Scores for Availability, Price, and Quality

Raters evaluated and scored each food measure based on its availability, price, and quality. Firstly, raters looked for healthier options for each food measure, as listed in Table 1. Then, they judged whether the healthier options were available and compared the price with regular options. Scores were given to each food measure based on availability of their healthier options and price, as listed in Table 2. Quality was only evaluated for fresh food, which included vegetables, fruits, meat and poultry, and seafood. Raters judged whether the quality of these fresh foods were acceptable or unacceptable. “Acceptable” (A) referred to peak condition, top quality, good color, and being fresh, firm, and clean. “Unacceptable” (UA) referred to being bruised, old looking, mushy, dry, or overripe, having dark sunken spots in irregular patches or cracked or broken surfaces, and signs of shriveling, mold or excessive softening. The rating was based on the majority (>50%) of vegetables, fruits, meat and poultry, or seafood. If it was difficult to decide whether to mark “A” or “UA”, “UA” was used, and a description was included in the comments.

### 2.2. Measurement Study

The adapted C-NEMS-S was tested in environment audits in 10 large-sized supermarkets (independent or chain, with areas more than 6000 m^2^) and 10 convenience stores (areas no more than 6000 m^2^) in February 2017. To assess inter-rater reliability, two raters entered the same store at the same time, with the store owners’ permission, and did the evaluations separately. The Research Ethics Committee of China Medical University approved the study ((2017) 055).

Two undergraduate students from China Medical University were recruited as raters. They were both junior students from preventive medicine and have taken courses on nutrition. Recruitment posters were posted on campus, and seven students signed up. These students took a test in nutrition, statistics, epidemiology, and environmental sciences, and the top two students were selected. Training for raters included taking the NEMS online courses [33], discussing with researchers about each food category and item in the C-NEMS-S, and studying a detailed operation guide on the C-NEMS-S.

### 2.3. Data Analysis

Both the intra-class correlation coefficient (ICC) and kappa coefficient were used to determine inter-rater reliability. The ICC was calculated based on composite score and availability score for each food category and item. An ICC less than 0.4 indicated poor inter-rater reliability and and ICC greater than 0.75 indicated good inter-rater reliability [34]. Kappa was calculated using the availability of the food item (yes/no). Kappa less than 0.4 indicated poor inter-rater reliability, a range between 0.4 to 0.6 represented middle inter-rater reliability, a range between 0.6 to 0.8 represented good inter-rater reliability, and greater than 0.8 indicated excellent inter-rater reliability [35].

The known-groups comparison method was used to determine construct validity [26]. If the scale was valid, the scores of the two groups with known differences would differ significantly. According to previous studies and the original NEMS-S, supermarkets and convenience stores were two groups with known differences: supermarkets were healthier than convenience stores [21,26,36,37]; healthier options and regular options were two groups with known differences with regard to price [21]. Therefore, we used scores acquired from the C-NEMS-S to test whether this instrument could also identify these differences. The Student’s *t* test was used to compare the total score between supermarkets and convenience stores. A nonparametric test was used to compare the availability between supermarkets and convenience stores and to compare prices between healthier options and regular options for each food measure. All data analyses were completed using SPSS Statistics version 20.0 (IBM Corporation, New York, NY, USA).

## 3. Results

### 3.1. The Adapted C-NEMS-S

Twelve measures were included in the C-NEMS-S (Supplementary Table S1: C-NEMS-S Questionnaire): grains, dry beans, starchy tubers, vegetables, fruits, seafood, meat and poultry, dietary oils, milk, bread, instant noodles, and beverages. Table 3 shows the food measurements and score dimensions.

### 3.2. Reliability

Table 4 shows the inter-rater reliability for the availability of each food measure. The intra-class correlation coefficient of availability had a composite score of 0.98. All food measures obtained a moderate or good ICC (ranging from 0.41 to 1.00). The kappa for each food measure ranged from 0.52 to 1.00. For price score and quality score, agreement between the two raters was very high, as shown in Supplement Table S2 and Supplement Table S3. Due to the low variance between different stores, ICC statistics were either too low or could not be calculated for price and quality.

### 3.3. Comparison between Chinese Supermarkets and Convenience Stores

As shown in Table 5, supermarkets scored significantly higher in total than convenience stores (*p* < 0.001). Table 6 shows the proportion of the food stores that provided healthier options for each food measure. Large-sized supermarkets had a significantly greater amount of healthier options available for all food measures (all 12 items were statistically significant (*p* < 0.05), except for sugar-free beverages). The results were consistent between rater 1 and rater 2.

### 3.4. Price Comparison between Healthier Options and Regular Options

Table 7 shows the price comparison between healthier options and regular options for each food measure. Healthier options cost more than regular options for grains, milk, bread, and instant noodles. The results were consistent between rater 1 and rater 2.

## 4. Discussion

The present study adapted and tested the first tool to measure the retail food environment in China. The original NEMS-S was adapted to the Chinese culture. The C-NEMS-S adapted in this study had high inter-rater reliability and was able to display the differences in healthy food availability between large-sized supermarkets and convenience stores, as well as the price differences between healthier options and regular options.

The NEMS-S is regarded as an observational measure or environmental auditing tool. The auditing ability of raters is critical to obtaining high reliability observations. For the original NEMS-S, the inter-rater kappa coefficient ranged from 0.83 to 1.00 [26]. For the Brazilian version of the NEMS-S, the inter-rater kappa coefficient ranged from 0.69 to 1.00, and the ICC raged from 0.75 to 1.00 [21]. The inter-rater reliability of the C-NEMS-S in the present study was only slightly lower than that of the original and the Brazilian version. Nonetheless, both the ICC and kappa coefficient were acceptable, ranging from moderate to high (0.41 to 1.00 for the ICC, 0.52 to 1.00 for the kappa coefficient) [34,35].

Compared with other food categories, milk, bread, instant noodles, and beverages had a lower ICC or kappa. This may be because packaged food categories occupied larger proportions of shelf space. In order to determine healthier options for these food categories, careful reading of food labels and packaging information is needed. Some raters may, therefore, fail to find healthier options for these food categories during this process. Though the ICC and kappa for these food categories were acceptable, future training programs, to teach raters how to measure in-store food environments, should pay more attention to these packaged food categories.

According to the results, large-sized supermarkets had a significantly greater amount of healthier options available for all food measures (all items were statistically significant (*p* < 0.05), except for sugar-free beverages) compared with convenience stores. The result was consistent with previous studies on differences between supermarkets and convenience stores. According to previous studies, supermarkets could provide healthier foods at reasonable prices [36,37]. For the original NEMS-S in the United States and the adapted NEMS-S in Brazil [21,26], supermarkets obtained higher scores on healthier food availability. Hence, supermarkets were hypothesized to score high in the C-NEMS-S, as well. According to the results in the present study, the C-NEMS-S obtained higher availability for all food measures in large-sized supermarkets. These findings in the present study support the ability of the C-NEMS-S to differentiate between supermarkets and convenience stores.

Except for meat and poultry, all healthier options cost more than regular options. This result indicates the ability of the measurement scoring to identify the differences between healthier options and regular options. In addition, this result also suggests that access to various food outlets might not be a problem for the food environment in China, but more attention should be paid to the consumer environment. The price discrepancy between healthier options and regular options may become a target for future policies aimed at improving the food environment in China.

There were several limitations in the present study. Firstly, dietary habit is different between northern China and southern China. The current validation survey of the C-NEMS-S was only conducted in Shenyang, a city in northern China. In addition, the field work happened in winter, so the seasonality of the food offerings could have played a role in influencing reliability testing and findings. However, the C-NEMS-S was developed based on guidelines suitable for the whole of China by referencing the Chinese Dietary Guidelines (2016) [29] and Chinese food categories in the China Food Composition handbook [28]. Food categories and species were selected based on a nation-wide perspective and were not limited to northern Chinese cuisine or limited by seasonality. With further validation surveys in cities in southern China and during different times of the year, the C-NEMS-S may be verified to be suitable for the whole of China. Secondly, a small sample size was used to conduct the environment audit in stores and to test the reliability of the C-NEMS-S. To cover more areas of China, a larger sample size is warranted in future validation studies. Thirdly, snacks were not included in the final version of the C-NEMS-S as a food measure. During evaluations, it was too difficult to determine low fat or low sugar healthier snacks from the large variety of snacks. This is a problem to be amended in updated versions of the C-NEMS-S in our future studies.

## 5. Conclusions

A Chinese version of the NEMS-S, which allowed for assessment of the food environment in China and potentially other Asian countries with similar food culture, was adapted and evaluated for China in the present study. The present study also implies that supermarkets could provide healthier food options compared to convenience stores in China. However, healthier options cost more than regular options, which may hinder the choice to purchase healthy foods and should be taken into consideration in future intervention studies or government food environment policies.

## Figures and Tables

**Table 1:** Definition of healthy foods and healthier options.

Food Measure	Definition of Healthier Option
Grains	Healthier option: Whole grains. Whole grains were selected as healthier options for grains due to their low glycemic index (GI) as recommended by the Chinese Dietary Guidelines and previous research [29].
Dry beans	Healthy food, as recommended by the Chinese Dietary Guidelines [29].
Starchy Tubers	Healthy food, as recommended by the Chinese Dietary Guidelines [29].
Vegetables	Healthy food, as recommended by the Chinese Dietary Guidelines [29].
Fruits	Healthy food, as recommended by the Chinese Dietary Guidelines [29].
Seafood	Healthy food, as recommended by the Chinese Dietary Guidelines [29].
Meat and Poultry	Healthier option: Lean meat options (tenderloin). Tenderloin contains less than 10% fat.
Dietary oils	Healthier option: Plant-based oils. Plant-based oils are recommended by the Chinese Dietary Guidelines as it is rich in Vitamin E, while animal oil has a higher proportion of saturated fat and cholesterol [29].
Milk	Healthier option: Low-fat milk. Packages are labeled as “low-fat”, which indicates fat content lower than 1.5 g/100 mL, and “no-fat”, which indicates fat content lower than 0.5%.
Bread	Healthier option: Whole wheat bread.
Instant noodles	Healthier option: Non-fried instant noodles
Beverages	Healthier option: Sugar-free beverages. Packages are labeled as “no-sugar”, which indicates sugar content lower than 1.5 g/100 g or 1.5 g/100 mL.

**Table 2:** Scoring Sheet for the Chinese version of the Nutritional Environment Measurement Survey (NEMS) for Stores.

Item	Availability of Healthier Option	Price	Quality
Grains	YES whole grains = 2 pts 2–3 species = 1 pt >3 species = 2 pts	Lower for whole grains = 2 pts Higher for whole grains = −1 pt	Not Applicable
Dry Beans	0 specie = 0 pt 1 species = 1 pt 2–3 species = 2 pts ≥4 species = 3 pts	Not Applicable	Not Applicable
Starchy Tubers	0 specie = 0 pt 1 species = 1 pt 2–4 species = 2 pts 5 species = 3 pts	Not Applicable	Not Applicable
Vegetables	0 specie = 0 pt 1–4 species = 1 pt 5–9 species = 2 pts ≥10 species = 3 pts	Not Applicable	25–49% acceptable = 1 pt 50–74% acceptable = 2 pts 75% + acceptable = 3 pts
Fruits	0 specie = 0 pt 1–4 species = 1 pt 5–9 species = 2 pts ≥10 species = 3 pts	Not Applicable	25–49% acceptable = 1 pt 50–74% acceptable = 2 pts 75% + acceptable = 3 pts
Seafood	0 specie = 0 pt 1–4 species = 1 pt 5–9 species = 2 pts ≥10 species = 3 pts	Not Applicable	25–49% acceptable = 1 pt 50–74% acceptable = 2 pts 75% + acceptable = 3 pts
Meat and Poultry	YES meat with less than 10% fat = 2 pts 2–3 species = 1 pt >3 species = 2 pts	Lower for lean meat = 2 pts Higher for lean meat = −1 pt	Not Applicable
Dietary oils	YES plant-based oils = 2 pts 2–3 species = 1 pt >3 species = 2 pts	Lower for plant-based oils = 2 pts Higher for plant-based oils = −1 pt	Not Applicable
Milk	YES low-fat milk = 2 pts Proportion (lowest fat to whole) >50% = 1 pt	Lower for low-fat milk = 2 pts Same for both = 1 pt Higher for low-fat milk = −1 pt	Not Applicable
Bread	YES whole wheat bread = 2 pts >2 species = 1 pt	Lower for whole wheat bread = 2 pts Higher for whole wheat bread = −1 pt	Not Applicable
Instant Noodles	YES non-fried instant noodles = 2 pts >2 species = 1 pt	Lower for non-fried instant noodles = 2 pts Higher for non-fried instant noodles = −1 pt	Not Applicable
Beverages	YES sugar-free carbonated beverages = 1 pt YES sugar-free lactose beverages = 1 pt YES sugar-free fruit juice = 1 pt YES sugar-free tea beverages = 1 pt YES sugar-free vegetable protein beverages = 1 pt YES sugar-free apple cider vinegar = 1 pt	Lower for sugar-free carbonated beverages = 2 pts Higher for sugar-free carbonated beverages = −1 pt Lower for sugar-free lactose beverages = 2 pts Higher for sugar-free lactose beverages = −1 pt Lower for sugar-free fruit juice = 2 pts Higher for sugar-free fruit juice = −1 pt Lower for sugar-free tea beverages = 2 pts Higher for sugar-free tea beverages = −1 pt Lower for sugar-free vegetable protein beverages = 2 pts Higher for sugar-free vegetable protein beverages = −1 pt Lower for sugar-free apple cider vinegar = 2 pts Higher for sugar-free apple cider vinegar = −1 pt	Not Applicable
	Availability Subtotal =	Price Subtotal =	Quality Subtotal =
Total Chinese version of the Nutritional Environment Measurement Survey (C-NEMS) Stores Score =			

POINT RANGES: Availability Subtotal: 0 to 48; Price Subtotal: −14 to 26; Quality Subtotal: 0 to 9; TOTAL NEMS SCORE RANGE: from −14 to 83.

**Table 3:** Food measures and score dimensions in the Chinese version of the Nutritional Environment Measurement Survey for Stores (C-NEMS-S).

Category of Food	Availability of Healthier Options		Price	Quality
	Healthier Option	Availability		
**Grains**	**Whole Grains**	X	X	
Dry beans ^1^	-	X		
Starchy Tubers ^1^	-	X		
Vegetables ^1^	-	X		X
Fruits ^1^	-	X		X
Seafood ^1^	-	X		X
Meat and Poultry	Lean meat options	X	X	X
Dietary oils	Plant-based oils	X	X	
Milk	Low-fat milk	X	X	
Bread	Whole wheat bread	X	X	
Instant noodles	Non-fried instant noodles	X	X	
Beverages	Sugar-free beverages	X	X	

^1^ Healthier food; no need for listing of healthier options. X Score dimensions.

**Table 4:** Inter-rater reliability of each food category based on availability.

Category of Food	ICC ^a^	Availability (Kappa) ^b^
Grains	0.99	1.00
Dry beans	0.99	1.00
Starchy Tubers	1.00	1.00
Vegetables	0.98	1.00
Fruits	0.91	0.80
Seafood	0.96	0.89
Meat and poultry	0.91	1.00
Dietary oils	0.93	0.54
Milk	0.63	0.52
Bread	0.75	0.60
Instant noodles	0.69	0.52
Beverages	0.41	0.60
Total	0.98	-

^a^ Intra-class correlation coefficient (ICC) was calculated based on availability score. ^b^ Kappa was calculated based on the availability of the food item (yes/no).

**Table 5:** C-NEMS-S total score by store type.

Rater	Supermarket		Convenience Store		*p*
	M ^a^	SD ^b^	M ^a^	SD ^b^	
Rater 1	31.80	2.44	1.90	2.28	<0.001
Rater 2	28.50	4.20	2.20	3.68	<0.001

^a^ M means the mean. ^b^ SD means the standard deviation.

**Table 6:** Availability (%) of healthier options by store type.

Category of Food	Large-Sized Supermarkets (*N* = 10)	Convenience Stores (*N* = 10)	*p*
**Rater 1**			
Whole grains	100	10	<0.001
Dry beans	90	10	0.002
Starchy Tubers	90	0	<0.001
Vegetables	100	0	<0.001
Fruits	100	10	<0.001
Seafood	80	0	0.002
Lean meat options	100	0	<0.001
Plant-based oils	100	20	0.002
Low-fat milk	80	20	0.023
Whole wheat bread	90	30	0.007
Non-fried instant noodles	60	0	0.023
Sugar-free beverages	80	30	0.063
**Rater 2**			
Whole grains	100	10	<0.001
Dry beans	90	10	0.002
Starchy Tubers	100	0	<0.001
Vegetables	100	0	<0.001
Fruits	100	10	<0.001
Seafood	100	0	<0.001
Lean meat options	80	0	0.002
Plant-based oils	100	20	0.002
Low-fat milk	60	0	0.023
Whole wheat bread	90	30	0.007
Non-fried instant noodles	60	0	0.023
Sugar-free beverages	80	30	0.063

**Table 7:** Price comparison between healthier option and regular option for each food measure.

Category of Food	Healthier Option		Regular Option		*p*
	M ^a^	SD ^b^	M ^a^	SD ^b^	
**Rater 1**					
Grains ^c^	6.19	2.27	2.45	0.09	<0.001
Meat and poultry ^c^	28.48	14.43	14.50	0.00	0.18
Milk ^d^	3.28	0.27	3.14	0.32	0.05
Bread ^e^	7.07	1.13	6.80	0.48	0.01
Instant noodles ^f^	3.37	0.25	2.50	0.15	<0.001
**Rater 2**					
Grains ^c^	5.94	2.46	2.62	0.32	<0.001
Meat and poultry ^c^	20.15	6.50	17.50	5.40	0.14
Milk ^d^	4.67	1.21	3.14	0.16	0.02
Bread ^e^	7.42	0.15	6.69	0.34	<0.001
Instant noodles ^f^	2.95	0.36	2.50	0.14	0.01

^a^ M means the mean. ^b^ SD means the standard deviation. ^c^ The unit is RMB/0.5 kg ^d^. The unit is RMB/250 mL. ^e^ The unit is RMB/ loaf. ^f^ The unit is RMB/ packet.

## Supplementary Materials

The following are available online at https://www.mdpi.com/1660-4601/16/5/782/s1, Table S1: Table s1. The C-NEMS-S. Table S2: Price scores by two raters. Table S3: Quality scores by two raters.
