# Supplementary material for: Adaptation and Validation of the Chinese Version of the Nutrition Environment Measurement Tool for Stores
Source: Int J Environ Res Public Health. 2019 Mar 4;16(5):782. doi: 10.3390/ijerph16050782 (PMC6427157; doi:10.3390/ijerph16050782)
Supplement: Supplementary file 1 [file ijerph-16-00782-s001.pdf]

Table S1. The C-NEMS-S.

Nutrition Environment Survey for Stores （NEMS-S）

Food Supplier Information

Rater ID：☐☐

○ Large sized supermarket

○ Convenience store

Store ID： ☐☐-☐☐-☐☐-☐☐

Date: ____Month ___ Day ____ Year

Start Time: __________ ○ AM ○ PM

End Time: __________ ○ AM ○ PM

Number of cash registers： ____

Comments:____

____

____

____

____

Nutrition Environment Survey for Stores （NEMS-S）
Cover Page

2006 Rollins School of Public Health, Emory University

2017Institute Health Science China Medical University

Not for reproduction or redistribution without permission

Nutrition Environment Survey for Stores（NEMS-S）

Measure #1：Grains

Rater ID: ☐☐ Store ID: ☐☐-☐☐-☐☐-☐☐☐

Date: ☐☐ Month ☐☐ Day ☐☐ Year ○Large Supermarket ○Convenience Store

Availability and Price

Produce Item	Size(g)	Available (Yes No N/A)	Price	Comments
**Healthier Option**				
1.Millet	☐☐	○ ○	☐☐.☐☐	________
**Alternate Items**				
2.Corn	☐☐	○ ○ ○	☐☐.☐☐	________
3.Others	☐☐	○ ○ ○	☐☐.☐☐	________

4.Total Crop Types ○1 ○2 ○3 ○4 ○5 ○6+

(Oats, rye, black rice, millet, corn, buckwheat, pearl barley, naked barley, sorghum, highland barely, coarse rice, millet bran)

Regular Option （Similar specifications and much healthier options can be bought）：

5.Rice (standard)	☐☐	○ ○	☐☐.☐☐	________
**Alternate Items**				
6.Refined Flour (standard)	☐☐	○ ○ ○	☐☐.☐☐	________

Nutrition Environment Survey for Stores（NEMS-S）

Measure #2：Dry Beans

Rater ID: ☐☐ Store ID: ☐☐-☐☐-☐☐-☐☐☐

Date: ☐☐Month ☐☐ Day ☐☐Year ○Large Supermarket ○Convenience Store

Availability and Price

Produce Item	Size(g)	Available<br>Yes No N/A	Price	Comments
**Healthier Option**				
1.Red Beans	☐☐	○ ○	☐☐. ☐☐	________
2.Mung Beans	☐☐	○ ○	☐☐. ☐☐	________
3.Pinto Bean	☐☐	○ ○	☐☐. ☐☐	________
4.Kidney Beans	☐☐	○ ○	☐☐. ☐☐	________
5.Broad Beans	☐☐	○ ○	☐☐. ☐☐	________
6.Peas (grain)	☐☐	○ ○	☐☐. ☐☐	________

7. Total Dry Beans Types：（Count of "Yes" Responses） ☐☐

Nutrition Environment Survey for Stores（NEMS-S）

Measure #3：Starchy Tubers

Rater ID: ☐☐ Store ID: ☐☐ - ☐☐ -☐☐-☐☐☐

Date: ☐☐ Month ☐☐ Day ☐☐ Year ○Large Supermarket ○Convenience Store

Availability and Price

Produce Item	Size(g)	Available<br>Yes No N/A	Price	Comments
**Healthier Option**				
1. Potato	☐☐	○ ○	☐☐. ☐☐	____________
2. Sweet Potato	☐☐	○ ○	☐☐. ☐☐	____________
3. Taro	☐☐	○ ○	☐☐. ☐☐	____________
4. Yam	☐☐	○ ○	☐☐. ☐☐	____________
5. Ginger	☐☐	○ ○	☐☐. ☐☐	____________

6. Total Starchy Tubers Types：（Count of "Yes" Responses）

Nutrition Environment Survey for Stores（NEMS-S）

Measure #4：Vegetables

Rater ID: ☐☐ Store ID: ☐☐-☐☐-☐☐-☐☐☐

Date: ☐☐Month ☐☐ Day ☐☐Year ○Large Supermarket ○Convenience Store

Availability and Price

Produce Item	Available<br>Yes No	Unit<br>(0.5 kilogram)	Quality<br>A UA	Comments

1.Root vegetables (radish, carrot, red skin radish, green-skin red-heart turnip , summer radish, etc)

a. Please judge	○ ○	☐☐	○ ○	______________
b. The number of root vegetable species ☐☐				
c. Price of carrot：______yuan/0.5 kilogram （the cheapest one）				

2.Fresh beans （mung bean sprout, soybean sprout, kidney bean (*jiadou wang*), snow pea, green soy bean, cowpea, pea (with pod), pea seeding, etc.）

a. Please judge	○ ○	☐☐	○ ○	______________
b. The number of fresh bean species ☐☐				
c. Price of kidney bean：______yuan/0.5 kilogram （the cheapest one）				

3.Solanaceous fruit vegetables (eggplant, tomato, bell pepper, cucumber, pumpkin, wax gourd, gourd, pumpkin, balsam pear, loofah, etc.)

a. Please judge	○ ○	☐☐	○ ○	______________
b. The number of solanaceous fruit vegetable species ☐☐				
c. Price of tomatoes：______yuan/0.5 kilogram （the cheapest one）				

4.Bulb vegetables （garlic, green onion, shallot, chive, onion, chives, blanched garlic leaves, garlic bolt, garlic sprout, leek, etc.）

a. Please judge	○ ○	☐☐	○ ○	______________
b. The number of fresh bean species ☐☐				
c. Price of bulb vegetables：______yuan/0.5 kilogram （the cheapest one）				

5. Tender stems, leaves, cauliflower vegetables （Chinese cabbage, bok choy, garden chrysanthemum, celery, coriander, romaine lettuce, rape, leaf lettuce, cauliflower, broccoli, cabbage, wild cabbage, spinach, bamboo shoots, potherb mustard, fennel, lettuce, etc.）

a. Please judge ○ ○ ☐☐ ○ ○ ______________

b. The number of tender stems, leaves, cauliflower vegetable species ☐☐

c. Price of Chinese cabbage：______yuan/0.5 kilogram （the cheapest one）

6. Aquatic vegetables （lotus foot, wild rice steam, etc.）

a. Please judge ○ ○ ☐☐ ○ ○ ______________

b. The number of aquatic vegetable species ☐☐

c. Price of lotus foot：______yuan/0.5 kilogram （the cheapest one）

7.Wild vegetables (fiddlehead, alfalfa, etc.)

a. Please judge ○ ○ ☐☐ ○ ○ ______________

b. The number of wild vegetable species ☐☐

c. Price of fiddlehead：______yuan/0.5 kilogram （the cheapest one）

8.Bacteria and algae vegetables （different kinds of mushrooms, edible tree fungi, kelp, laver, etc.）

a. Please judge ○ ○ ☐☐ ○ ○ ______________

b. The number of bacteria and algae vegetable species ☐☐

c. Price of fiddlehead：______yuan/0.5 kilogram （the cheapest one）

9.Total Vegetables Types： （Count of “Yes” Responses）☐☐

Nutrition Environment Survey for Stores（NEMS-S）

Measure #5：Fruits

Rater ID: ☐☐ Store ID: ☐☐-☐☐-☐☐-☐☐☐

Date: ☐☐Month ☐☐ Day ☐☐Year ○Large Supermarket ○Convenience Store

Availability and Price

Produce Item	Available Yes No	Unit (0.5 kilogram)	Quality A UA	Comments

1.Pip fruits (apples, various pears, hawthorn, crab apple, etc.)

a. Please judge	○ ○	☐☐	○ ○	______________
b. The number of pip fruit species		☐☐		
c. Price of apples：______yuan/0.5 kilogram （the cheapest one）				

2. Stone fruits (peach, apricot, plum, cherry, jujube (fresh/dried), etc.）

a. Please judge	○ ○	☐☐	○ ○	______________
b. The number of stone fruit species		☐☐		
c. Price of peaches：______yuan/0.5 kilogram （the cheapest one）				

3.Soft fruits (various grapes, raisin, strawberry, kiwi fruit, pomegranate, persimmon, dried persimmon, sea-buckthorn, mulberry, common fig, etc.)

a. Please judge	○ ○	☐☐	○ ○	______________
b. The number of soft fruit species		☐☐		
c. Price of grapes：______yuan/0.5 kilogram （the cheapest one）				

4.Citrus fruits （orange, various citrus, pomelo, lemon, etc.）

a. Please judge	○ ○	☐☐	○ ○	______________
b. The number of citrus fruit species		☐☐		
c. Price of oranges：______yuan/0.5 kilogram （the cheapest one）				

5.Subtropical and tropical fruits (banana, pineapple, mango, litchi, longan, jackfruit, durian, red bayberry, pawpaw, coconut, guava, loquat, star fruit, etc.)

a. Please judge ○ ○ □□ ○ ○ ________

b. The number of subtropical and tropical fruit species □□

c. Price of bananas：______yuan/0.5 kilogram （the cheapest one）

6.Melon（watermelon, honeydew melon, muskmelon, cantaloupe, golden melon, etc.)

a. Please judge ○ ○ □□ ○ ○ ________

b. The number of melon and fruit species □□

c. Price of watermelons：______yuan/0.5 kilogram （the cheapest one）

7.Total Fruits Types：（Count of “Yes” Responses） □□

Nutrition Environment Survey for Stores（NEMS-S）
Measure #6：Seafood

Rater ID: ☐☐　　Store ID: ☐☐-☐☐-☐☐-☐☐☐

Date: ☐☐Month ☐☐ Day ☐☐Year　　○Large Supermarket　○Convenience Store

Availability and Price

Produce Item	Available Yes No	Unit (0.5 kilogram)	Quality A UA	Comments

1.Fishs (hair tail, yellow croaker, grass carp, Spanish mackerel, black carp, carp, crucian, sardine, etc.)

a. Please judge	○ ○	☐☐	○ ○	______________
b. The number of fish species		☐☐		
c. Price of grass carp：______yuan/0.5 kilogram （the cheapest one）				

2.Shrimps (prawn, river shrimp, brine shrimp, etc.)

a. Please judge	○ ○	☐☐	○ ○	______________
b. The number of shrimp species		☐☐		
c. Price of shrimps：______yuan/0.5 kilogram （the cheapest one）				

3.Crabs (sea crab, river crab, etc.)

a. Please judge	○ ○	☐☐	○ ○	______________
b. The number of crab species		☐☐		
c. Price of crabs：______yuan/0.5 kilogram （the cheapest one）				

4.Shellfishs (abalone, clam, fresh oyster, oyster, fan shell, razor clam, various nails, etc.)

a. Please judge	○ ○	☐☐	○ ○	______________
b. The number of shellfish species		☐☐		
c. Price of shellfishs：______yuan/0.5 kilogram （the cheapest one）				

5.Total Seafood Types：（Count of “Yes” Responses） ☐☐

Nutrition Environment Survey for Stores（NEMS-S）

Measure #7：Meat and Poultry

Rater ID: ☐☐ Store ID: ☐☐-☐☐-☐☐-☐☐☐

Date: ☐☐ Month ☐☐ Day ☐☐ Year ○ Large Supermarket ○ Convenience Store

Availability and Price

Produce Item	Available Yes	Available No	Price(yuan)	Unit(0.5 kilogram)	Comments
**Healthier Option**					
1.Pork with less than 10% fat(tenderloin)	○	○	☐☐.☐☐	☐☐	________
**Alternate Items**					
2.Lean meat	○	○	☐☐.☐☐	☐☐	________
3. Chicken（Free-range, domestic, breast）	○	○	☐☐.☐☐	☐☐	________
4. Beef（Lean）	○	○	☐☐.☐☐	☐☐	________
5. Mutton（Tenderloin/lamb leg）	○	○	☐☐.☐☐	☐☐	________

6. Total meat/chicken/beef/mutton (tenderloin/lamb leg) with less then 10% fat types

1 ○ 2 ○ 3 ○ 4 ○ 5 ○ 6+ ○

Produce Item	Available Yes	Available No	Price(yuan)	Unit(0.5 kilogram)	Comments
**Regular Option**					
7.Pork with more than 10% fat(pork belly)	○	○	☐☐.☐☐	☐☐	________
**Alternate Items**					
8.Haunch pork	○	○	☐☐.☐☐	☐☐	________
9.Chicken (Broiler, drumstick, chicken wing）	○	○	☐☐.☐☐	☐☐	________
10.Rib meat	○	○	☐☐.☐☐	☐☐	________
11.Mutton(frozen)	○	○	☐☐.☐☐	☐☐	________

Nutrition Environment Survey for Stores（NEMS-S）

Measure #8：Dietary oils

Rater ID: ☐☐ Store ID: ☐☐-☐☐-☐☐-☐☐☐

Date: ☐☐Month ☐☐ Day ☐☐Year ○Large Supermarket ○Convenience Store

Availability and Price

Produce Item	Available (Yes / No)	Price(yuan)	Unit(0.5 kilogram)	Comments
**Healthier Option**				
1.Soybean oil	○ ○	☐☐.☐☐	☐☐	________
**Alternate Items**				
2.Colza oil	○ ○	☐☐.☐☐	☐☐	________
3.Corn oil	○ ○	☐☐.☐☐	☐☐	________
4.Sunflower oil	○ ○	☐☐.☐☐	☐☐	________
5.Olive Oil	○ ○	☐☐.☐☐	☐☐	________

6. Total Plant-based Oil Types

1 ○ 2 ○ 3 ○ 4 ○ 5 ○ 6+ ○

Produce Item	Available (Yes / No)	Price(yuan)	Unit(0.5 kilogram)	Comments
**Regular Option**				
7.Lard	○ ○	☐☐.☐☐	☐☐	________
**Alternate Items**				
8.Beef tallow	○ ○	☐☐.☐☐	☐☐	________
9.Mutton tallow	○ ○	☐☐.☐☐	☐☐	________
10.Butter	○ ○	☐☐.☐☐	☐☐	________

Nutrition Environment Survey for Stores（NEMS-S）

Measure #9：Milk

Rater ID: ☐☐ Store ID: ☐☐-☐☐-☐☐-☐☐☐

Date: ☐☐Month ☐☐ Day ☐☐Year ○Large Supermarket ○Convenience Store

Availability

1.Is low fat milk available? ○ Yes ○ No

Comments ____________________

2.Shelf Space（Measure only if low fat milk is available)

Type	220ml	250ml	1L
a.low fat	☐☐	☐☐	☐☐
b.whole	☐☐	☐☐	☐☐

Comments ____________________

**Compare**	**Price(yuan)**	**Comments**
1.Whole Milk，220ml	☐.☐ ☐☐	____________________
2.Whole Milk，250ml	☐.☐ ☐☐	____________________
3.Whole Milk，1L	☐.☐ ☐☐	____________________
4.Low Fat Milk，240ml（Lowest-fat Milk Available）	☐.☐ ☐☐	____________________
5.Low Fat Milk，250ml（Lowest-fat Milk Available）	☐.☐ ☐☐	____________________
6.Low Fat Milk，1L（Lowest-fat Milk Available）	☐.☐ ☐☐	____________________

Nutrition Environment Survey for Stores（NEMS-S）

Measure #10：Bread

Rater ID: ☐☐ Store ID: ☐☐-☐☐-☐☐-☐☐☐

Date: ☐☐ Month ☐☐ Day ☐☐ Year ○Large Supermarket ○Convenience Store

Availability and Price

Produce Item	Available Yes	Available No	Loaf price(yuan)	Unit(0.5 kilogram)	Comments
**Healthier Option**					
1.Whole Grain Bread (100% whole wheat bread and whole grain bread)	○	○	☐☐.☐☐	☐☐	________
**Alternate Items**					
2 Whole Grain Bread (Other brands)	○	○	☐☐.☐☐	☐☐	________
3.Steamed bun (Standard flour)	○	○	☐☐.☐☐	☐☐	________

4. Total100%Whole Grain Bread Types of All Brands

1 ○ 2 ○ 3 ○ 4 ○ 5 ○ 6+ ○

Produce Item	Available Yes	Available No	Loaf price(yuan)	Unit(0.5 kilogram)	Comments
**Regular Option: White bread （bread made with refined flour）**					
5.White bread (Other brands)	○	○	☐☐.☐☐	☐☐	________
**Alternate Items**					
6.White bread (Other brands)	○	○	☐☐.☐☐	☐☐	________
7.White bread (Standard flour)	○	○	☐☐.☐☐	☐☐	________

Nutrition Environment Survey for Stores（NEMS-S）

Measure #11：Instant Noodles

Rater ID: ☐☐ Store ID: ☐☐-☐☐-☐☐-☐☐☐

Date: ☐☐ Month ☐☐ Day ☐☐ Year ○Large Supermarket ○Convenience Store

Availability and Price

Produce Item	Available Yes No	Loaf price(yuan)	Price(yuan)	Comments
**Healthier Option**				
1.Fried-free instant noodles	○ ○	☐☐.☐☐	☐☐	________
**Alternate Items**				
2. ________	○ ○	☐☐.☐☐	☐☐	________

3. Total fried-free instant noodle of all brands

1 ○ 2 ○ 3 ○ 4 ○ 5 ○ 6+ ○

**Regular Option: White bread （select most comparable size to healthier option available)**				
4. Regular instant Noodle (classic taste)	○ ○	☐☐.☐☐	☐☐	________
**Alternate Items**				
5. ________	○ ○	☐☐.☐☐	☐☐	________

Nutrition Environment Survey for Stores（NEMS-S）

Measure #12：Bvevrages

Rater ID: ☐☐ Store ID: ☐☐- ☐☐ -☐☐-☐☐☐

Date: ☐☐Month ☐☐ Day ☐☐Year ○Large Supermarket ○Convenience Store

Availability and Price

Produce Item	Available Yes	Available No	Whether cheaper than common drinks of the same brand Yes	Whether cheaper than common drinks of the same brand No	Comments
1.Sugar free lactic acid drinks	○	○	○	○	________________
2.Sugar free milk beverage	○	○	○	○	________________
3.Sugar free juice	○	○	○	○	________________
4.Sugar free tea drinks	○	○	○	○	________________
5.Sugar free plant protein beverage	○	○	○	○	________________
6.Sugar free apple vinegar	○	○	○	○	________________

7.Total free sugar drink types：（Count of “Yes” Responses）________________

Table S2. Price scores by two raters.

	Grains		Meat and Poultry		Milk		Bread		Instant Noodles		Beverages	
	Rater1	Rater2	Rater1	Rater2	Rater1	Rater2	Rater1	Rater2	Rater1	Rater2	Rater1	Rater2
1	−1.00	−1.00	−1.00	−1.00	1.00	1.00	−1.00	−1.00	–	–	−1.00	−2.00
2	−1.00	−1.00	2.00	–	1.00	−1.00	−1.00	−1.00	–	–	−1.00	−2.00
3	−1.00	−1.00	−1.00	2.00	−1.00	2.00	−1.00	−1.00	–	–	−1.00	−1.00
4	−1.00	−1.00	−1.00	−1.00	−1.00	−1.00	−1.00	−1.00	–	–	−1.00	−1.00
5	−1.00	−1.00	−1.00	−1.00	–	–	−1.00	−1.00	−1.00	−1.00	−1.00	−2.00
6	−1.00	−1.00	−1.00	−1.00	−1.00	−1.00	2.00	−1.00	−1.00	–	−1.00	−1.00
7	−1.00	−1.00	−1.00	−1.00	−1.00	2.00	−1.00	−1.00	−1.00	−1.00	−1.00	−1.00
8	−1.00	−1.00	−1.00	2.00	−1.00	−1.00	−1.00	−1.00	−1.00	−1.00	–	–
9	−1.00	−1.00	−1.00	2.00	1.00	−1.00	–	–	–	–	−1.00	2.00
10	−1.00	−1.00	−1.00	−1.00	1.00	1.00	−1.00	−1.00	−1.00	−1.00	–	–
11	–	–	–	–	1.00	–	–	−1.00	–	–	–	–
12	–	–	–	–	–	–	−1.00	–	–	–	–	–
13	–	–	–	–	–	–	–	–	–	–	–	–
14	–	–	–	–	–	–	–	–	–	–	–	–
15	–	–	–	–	–	–	–	–	–	–	–	–
16	–	–	–	–	–	–	–	–	–	–	–	−1.00
17	–	–	–	–	–	–	–	–	–	–	−1.00	–
18	–	–	–	–	–	–	–	–	–	–	–	−1.00
19	–	–	–	–	–	−1.00	–	–	–	–	−1.00	–
20	−1.00	−1.00	–	–	−1.00	1.00	–	–	–	–	−1.00	2.00

Table S3. Quality scores by two raters.

	Vegetables		Fruits		Seafood	
	**Rater1**	**Rater2**	**Rater1**	**Rater2**	**Rater1**	**Rater2**
**1**	**3.00**	**3.00**	**3.00**	**3.00**	**3.00**	**3.00**
**2**	**3.00**	**3.00**	**3.00**	**3.00**	**3.00**	**3.00**
**3**	**3.00**	**3.00**	**3.00**	**3.00**	**3.00**	**3.00**
**4**	**3.00**	**3.00**	**3.00**	**3.00**	**3.00**	**3.00**
**5**	**3.00**	**3.00**	**3.00**	**3.00**	**3.00**	**3.00**
**6**	**3.00**	**3.00**	**3.00**	**3.00**	**3.00**	**3.00**
**7**	**3.00**	**3.00**	**3.00**	**3.00**	**3.00**	**3.00**
**8**	**3.00**	**3.00**	**3.00**	**3.00**	**3.00**	**3.00**
**9**	**3.00**	**3.00**	**3.00**	**3.00**	–	–
**10**	**3.00**	**3.00**	**3.00**	**3.00**	**3.00**	**3.00**
**11**	–	–	–	–	–	–
**12**	–	–	–	–	–	–
**13**	–	–	–	–	–	–
**14**	–	–	–	–	–	–
**15**	–	–	–	–	–	–
**16**	–	–	–	–	–	–
**17**	–	–	–	–	–	–
**18**	–	–	–	**3.00**	–	–
**19**	–	–	**3.00**	–	–	**−1.00**
**20**	–	–	–	–	**−1.00**	**1.00**
